# M2 macrophage polarization: a potential target in pain relief

**DOI:** 10.3389/fimmu.2023.1243149

**Published:** 2023-08-29

**Authors:** Wenjing Zhao, Lulin Ma, Daling Deng, Tianhao Zhang, Linlin Han, Feng Xu, Shiqian Huang, Yuanyuan Ding, Xiangdong Chen

**Affiliations:** ^1^Department of Anesthesiology, Union Hospital, Tongji Medical College, Huazhong University of Science and Technology, Wuhan, China; ^2^Institute of Anesthesia and Critical Care Medicine, Union Hospital, Tongji Medical College, Huazhong University of Science and Technology, Wuhan, China; ^3^Key Laboratory of Anesthesiology and Resuscitation, Huazhong University of Science and Technology, Ministry of Education, Wuhan, China

**Keywords:** pain, M2 macrophage, macrophage polarization, pain mechanism, pain treatment

## Abstract

Pain imposes a significant urden on patients, affecting them physically, psychologically, and economically. Despite numerous studies on the pathogenesis of pain, its clinical management remains suboptimal, leading to the under-treatment of many pain patients. Recently, research on the role of macrophages in pain processes has been increasing, offering potential for novel therapeutic approaches. Macrophages, being indispensable immune cells in the innate immune system, exhibit remarkable diversity and plasticity. However, the majority of research has primarily focused on the contributions of M1 macrophages in promoting pain. During the late stage of tissue damage or inflammatory invasion, M1 macrophages typically transition into M2 macrophages. In recent years, growing evidence has highlighted the role of M2 macrophages in pain relief. In this review, we summarize the mechanisms involved in M2 macrophage polarization and discuss their emerging roles in pain relief. Notably, M2 macrophages appear to be key players in multiple endogenous pathways that promote pain relief. We further analyze potential pathways through which M2 macrophages may alleviate pain.

## 1 Introduction

Pain can be induced by tissue damage or inflammation invasion. While most patients experience gradual pain relief as wounds heal and inflammation subsides, some cases may progress to chronic pain. For example, 30% of patients with chemotherapy-induced peripheral neuropathy (CIPN) still experience pain six months after chemotherapy (1). Additionally, approximately 10% of patients undergoing surgical procedures suffer from chronic pain that cannot be effectively managed (2). Chronic pain, an unpleasant subjective sensation, has significant negative effects on physical and mental health, affecting about 30% of patients worldwide (3). Presently, opioids and non-steroidal anti-inflammatory drugs (NSAIDs) are the main treatments for pain. However, opioids are increasingly stigmatized due to their addictive, tolerant, and analgesic side effects (4), while NSAIDs may lead to gastrointestinal and cardiovascular reactions (5). Emerging studies also suggest that NSAIDs during the acute phase may contribute to the development of chronic pain (6). Therefore, pain management remains unsatisfactory. While extensive preclinical studies have investigated the mechanisms of pain development (7–10), mechanisms of pain relief have received less attention. Understanding pain relief offers an alternative perspective for pain treatment, that is, to intervene in the mechanism of pain relief, enhance this mechanism, thereby alleviating pain.

Macrophages are an important component of the body’s immune system. Numerous studies suggest that immune cells play crucial regulatory roles in pain development (10–12). Under various stimuli, macrophages differentiate into different phenotypes, each exhibiting distinct characteristics and functions, and playing diverse regulatory roles in physiological and pathological processes. Following peripheral nerve injury, macrophages can transform into the M1 phenotype, producing inflammatory factors that promote neuropathic pain (13). In contrast, transitioning into the M2 phenotype can inhibit inflammation, promote tissue healing, and subsequently relieve neuropathic pain. While the promoting role of M1 macrophages in pain has been extensively summarized, this review emphasizes the role of M2 macrophages in pain relief (14, 15). In this review, we emphasize the role of M2 macrophages in the process of pain relief. Notably, many pain relief measures function by promoting the polarization of M2 macrophages. Consequently, we focus on elucidating how M2 macrophages contribute to pain relief. This review aims to provide evidence for pain relief through the regulation of M2 macrophages and to present novel ideas for pain treatment.

## 2 Pain relief is an active process

Pain relief, traditionally considered a passive process, linked to the subsiding of inflammation, tissue healing, and dissipation of pain-inducing factors, is now being recognized as an active and dynamic phenomenon. The nervous system does not easily revert to its baseline state after nociception occurs, instead transitioning to a state of “latent sensitization” or “hyperalgesic priming”, leading to more severe pain upon reexposure to the stimulus (16, 17). Recent research indicates that pain relief is an active and dynamic process, distinct from pain maintenance. In the case of lower back pain, patients who experience pain relief demonstrate significant alterations in over 5500 genes in their peripheral blood during the relief period compared to the acute phase. Conversely, patients whose pain remains unresolved do not show significant changes in gene expression levels before and after pain (6). A prolactin induced mouse pain model revealed significant changes in gene expression in the dorsal root ganglia (DRG) and hind paw tissues of both female and male mice during the relief period (18). These findings highlight the involvement of numerous active biological processes that occur during pain relief. Moreover, recent evidence suggests that the secretion of specialized pro-resolving mediators (SPMs) such as lipoxins, maresins, resolvins, protectins, etc., alongside the phenotypic conversion of immune cells (including macrophages, T cells, and neutrophils), play pivotal roles in mediating inflammation resolution and pain relief (19).Targeting these resolution pathways offers a new perspective on pain treatment, holding promise in effectively managing acute pain and potentially preventing chronic pain.

## 3 Regulation of M2 macrophage polarization

Macrophages are key immune cells involved in the innate immune response. Their activation plays a critical role in the inflammatory response, tissue recovery and homeostasis (20). Macrophages can be classified into M1 macrophages with a pro-inflammatory phenotype and M2 macrophages with an anti-inflammatory phenotype in the latest classification. M2 macrophages are further divided into M2a (IL-4/IL-13), M2b (immune complexes and Toll-like-receptor or IL‐1R agonists), and M2c (IL‐10) (21, 22). Macrophages do not strictly exist in a bipolar state; rather, they constantly switch and transition between M1 and M2 states, suggesting the possibility of an intermediate state with both pro-inflammatory and anti-inflammatory phenotypes (23). In this study, we utilized known macrophage markers that are altered during polarization to distinguish between M1 and M2 macrophages. Specifically, M1 macrophages were identified by overexpression of CD80, CD86, iNOS, STAT-1 and MHC-II (24), while M2 macrophages were characterized by the expression of CD200R, CD206, CD163, Arg-1, STAT-3, and IL-10 (25). Several signaling pathways and metabolic reprogramming are involved in regulating macrophage polarization. As this paper primarily focuses on M2 macrophages, the following content will summarize the mechanisms involved in M2 macrophage polarization.

### 3.1 JAK/STATs signaling pathway

Janus kinase (JAK) is a tyrosine kinase with four types, JAK1, JAK2, JAK3, and TYK2. The signal transducer and activator of transcriptions (STATs) include six isoforms, STAT1-6. The JAK/STATs signaling pathway is activated during M2 macrophage polarization and plays a role in regulating the transcription of related genes. For example, M2 macrophage polarization induced by IL4 is inextricably linked to the activation of the JAK1-STAT6 signaling pathway (26), leading to the activation of M2-like genes, such as YM1, Arg1, Fizz1, IL-10, and MGL1 (27). Inhibition of the JAK2-STAT3 signaling pathway results in M1 macrophage polarization, and activation of the JAK2-STAT3 signaling pathway promotes M2 polarization (28). Activation of the JAK1-STAT1 signaling pathway facilitates macrophage conversion to the M1-type (29), whereas STAT3 activation inhibits the expression of STAT1, thereby suppressing its role in mediating M1 macrophage polarization while enhancing M2 macrophage polarization (30). Overall, the activation of STAT3 and STAT6 promotes the transcription of M2 macrophage-associated genes, leading to metabolic and functional alterations in M2 macrophages and enhancing their anti-inflammatory effects.

### 3.2 TGF-β signaling pathway

Transforming growth factor-β (TGF-β) is a multifunctional cytokine that plays an important role in the polarization process of M2 macrophages. It not only polarizes monocyte to M2 macrophages but also repolarizes LPS-induced M1 macrophages into M2 macrophages (31). This process involves the binding of TGF-β to type 2 TGF-β receptors and the recruitment of type 1 TGF-β receptors, followed by the activation of Smad2/3. The activated Smad2/3 forms a heterodimer with Smad4 and enters the nucleus, where it can subsequently regulate the expression of M2 macrophage-related genes and facilitate the process of M2 macrophage polarization (32). Additionally, TGF-β can also promote M2 macrophage polarization through non-Smad pathways (33). In summary, TGF-β plays an important role in regulating M2 macrophage polarization and modulating immune responses.

### 3.3 PPARγ signaling pathway

Peroxisome proliferator-activated receptor gamma (PPARγ) is a nuclear receptor that directly regulates the transcription of target genes upon ligand binding in the nucleus, thereby influencing cellular functions. Activation of PPARγ increases gene expression of Mrc1 and Arg1 while decreasing gene expression of iNOS, promoting the conversion to the M2 type. Conversely, inhibiting of PPARγ suppresses M2 macrophages and increases the proportion of M1 macrophages (34). He et al. conducted a comprehensive analysis of dynamic changes in cell signaling and metabolism during macrophage polarization using quantitative time-course proteomics and phosphoproteomics. They also identified pharmacological inhibitors that can prevent M2-type macrophage polarization. The study results indicated that PPARγ/retinoic acid plays a crucial role in inducing the polarization of M2 macrophages. Additionally, the activation of mitogen-activated protein kinase was found to be necessary for this process to occur (35).

### 3.4 Metabolic regulation of M2 macrophage polarization

In response to a series of external cues, macrophages undergo a significant switch in their metabolic pathways. Numerous studies have demonstrated that glycolysis is significantly enhanced in M1 macrophages, while M2 macrophages rely more on fatty acid oxidation and oxidative phosphorylation (OXPHOS) (36). Pyruvate generated from glycolysis can enter OXPHOS, but in M2 macrophages, OXPHOS can occur independently of this pathway. Inhibiting glycolysis does not impact M2 macrophage polarization, as long as OXPHOS remains functional (37). Fatty acid oxidation is fueled by the breakdown of triglycerides. M2 macrophages obtain triglyceride-rich lipid droplets from adipose tissue *via* CD36, which they can take up and catabolize for energy. Additionally, M2 macrophages are capable of *de novo* triglyceride synthesis (38). Lysosomal acid lipase is involved in the breakdown of triglycerides and the release of fatty acids. M2 macrophages express lysosomal acid lipase at a much higher level than M1 macrophages (38). The oxidative metabolism of fatty acids contributes to the expression of M2 macrophage characteristic genes such as Arg-1 and IL-10 (39). Components involved in triglyceride catabolism and fatty acid oxidation can influence M2 macrophage polarization. For instance, orlistat, a lipolysis inhibitor, has been shown to hinder the polarization of M2 macrophages (38). Dioscin has been found to promote M2 macrophage polarization by enhancing fatty acid catabolism through the mTORC2/PPAR-γ signaling pathway. However, the fatty acid catabolism inhibitor etomoxir can reverse the pro-M2 macrophage polarizing effect of dioscin. Glutamine, an important amino acid metabolite, plays an important role in cellular metabolism and is vital for M2 macrophage polarization. Deprivation of glutamine in bone marrow-derived macrophages impairs M2 macrophage polarization (40). Glutamine is catabolized by glutamate dehydrogenase 1 to produce α-ketoglutarate (αKG), which induces M2 macrophage polarization and is regulated by SENP1-Sirt3 (41). The catabolism of glutamine generates αKG, an intermediate product of the tricarboxylic acid cycle. αKG can enter the mitochondrial OXPHOS metabolism pathway to produce ATP and also plays a role in histone modification. Histone modification involving H3K27 trimethylation is a common regulatory mechanism for gene expression. αKG can decrease H3K27 trimethylation in the nucleus, resulting in the upregulation of genes associated with M2 polarization (41). Additionally, αKG is also involved in fatty acid oxidation (42), indirectly affecting M2 macrophage polarization. Thus, regulating macrophage metabolism is a way of modulating the polarization of macrophages towards the M2 type.

## 4 The role of M2 macrophages in pain relief

Following inflammatory insults or tissue injury, macrophages derived from circulating monocytes infiltrate into tissues. Both infiltrating and tissue-resident macrophages become activated and accumulate around damaged sites and the DRG of nociceptive neurons. Recent research indicates that while bone marrow-derived macrophages increase in number at the DRG following nerve injury, the majority of macrophages in this area are of tissue origin (43). Macrophages in the DRG, but not those around the injured-site, are believed to be the primary contributors to the initiation and development of neuropathic pain and inflammatory pain (13, 44, 45). However, Shepherd et al. hold the opposite view, suggesting that macrophages around the injured-site play a more vital role in pain compared to those in the DRG (46). The exact contribution of macrophages in the DRG versus those at the injury site, or both, in pain, remains unclear. However, we are more inclined to believe that macrophages in the DRG play a role in regulating pain because macrophages at the site of injury often exhibit inconsistent behaviors in the development of pain (45). Nevertheless, the dialogue between macrophages and neurons plays a regulatory role in the occurrence and development of pain.

When the body is damaged, macrophages are activated and polarized to M1. M1 macrophages release a large number of inflammatory factors, such as IL6, IL-1β, TNF-α, IGF-1, and so on. These inflammatory factors play critical roles in increasing the excitability of nociceptive neuron, thereby promoting the development of pain (15). In the first few days after injury, the injury or inflammation region and corresponding DRG are dominated by M1 macrophages, whereas several days later, during the resolution of pain, the level of M1 macrophages returns to the baseline level, and M2 macrophages take over (13, 45, 47, 48). Under normal conditions, DRG neurons are surrounded by satellite glial cells with a narrow gap of only 20 nm (49). After nerve injury, the gap between neurons and satellite glial cells increases, and M2 macrophages undergo enlargement and develop an astral shape. This shape allows M2 macrophages to penetrate between neurons and glial cells, a phenomenon observed both in injured and uninjured neurons. The neurons in close contact with M2 macrophages are protected from neuronal death (43).On the seventh day after the injury, a significant increase in M2 macrophages was observed in the DRG on the injured side compared to the contralateral side, coinciding with pain relief. However, there was no significant difference in the number of M1 macrophages between the two sides (43). A similar phenomenon of macrophage changes over time after an injury can also be found in the spinal cord (50).

Additionally, there is abundant direct evidence demonstrating the crucial role of M2 macrophages in pain relief, which is summarized in **Table 1**. MRC1^+^ macrophages, markers of M2 macrophages in the spinal cord, proliferate during pain relief, highlighting their importance in pain relief (50). However, compared to those in superficial injury, the expansion of spinal M2 macrophages in nerve injury is noticeably blunted, accompanied by delayed pain relief (50). Although no research study has directly addressed whether this phenomenon still exists in the DRG or at the injured-site, other evidence supports the importance of M2 macrophages during the pain relief. Michiel et al. discovered that diphtheria toxin, which induced the complete depletion of monocytes and macrophages, delayed the recovery of acute inflammatory pain induced by Carrageenan. Intrathecal injection of M2 macrophages but not M1 macrophages can reverse this situation (47). Moreover, intrathecal injection of M2 macrophages can also resolve the persistent inflammatory pain induced by monoiodoacetate in the osteoarthritis (OA) model (13). Selectively depleting M2 macrophages by intrathecal injection of m-clodrosome was enough to delay the recovery of the mechanical pain threshold decreased by cisplatin (52). Additionally, intrathecal injection of M2-like bone marrow derived macrophages can relieve spared nerve injury (SNI) -induced pain (51). These are direct evidence of the involvement of M2 macrophages in pain relief.

**Table 1:** The role of M2 macrophages in different pain models.

Region	Pain model	Species	Intervene	Effect	Reference
DRG	OA	Mice	Intrathecal injection of M2 macrophages	Mechanical hypersensitivity↓	(13)
Joint	K/BxN serum transfer model	Mice	–	The number of M2 macrophages at the joint does not consistently correlate with pain progression	(45)
DRG	Carrageenan-induced inflammatory pain	Mice	Intrathecal injection of M2 macrophages	The delayed pain relief caused by macrophage depletion is rescued.	(47)
Spinal cord	SNI	Mice	–	The expansion of spinal MRC1 macrophages is obviously blunted.	(50)
DRG	SNI	Mice	Intrathecal injection of M2-like bone marrow derived macrophages	M2 macrophages↑ Mechanical hypersensitivity↓	(51)
DRG	CIPN	Mice	Intrathecal injection of m-clodrosome	M2 macrophages↓ Resolution of mechanical allodynia↓	(52)
Injured nerve	CCI	Mice	Injection of IL-4 (200 ng) at the injured site from day 14 to day 21 after CCI	M2 macrophages↑ Mechanical hypersensitivity↓	(53)
Hind paw	Zymosan-induced inflammatory pain	Mice	Systemic injection of the MΦ toxin clodronate	Pain relief was delayed, which can be rescued by the transplantation of normal macrophages.	(54)

↑ means rise or promotion.↓ means decline or inhibition.

M2 macrophages may be common participants in multiple endogenous pathways that promote pain relief. T cells, SPMs, and IL-4 all play crucial roles in the natural course of pain relief (55–57). The polarization of M2 macrophages is essential in these processes. These factors regulate the polarization of M2 macrophages through various mechanisms, thereby promoting pain relief. In cisplatin-induced pain relief, the secretion of IL-13 by CD8^+^ T cells promotes the conversion of macrophages to the M2 phenotype. Blocking IL-13 signaling from T cells inhibits this conversion process and prevents pain relief (52). In the chronic constriction injury (CCI) model, a single injection of IL-4 at the injured nerve resulted in pain relief through the release of opioid peptides from macrophages located at the site of damage. However, this effect is short-lived and the macrophages at the site of damage remain predominantly of the M1 phenotype (58). Prolonged application of IL-4 to the injured nerve over several days leads to a shift of macrophages from a pro-inflammatory M1 phenotype to an anti-inflammatory M2 phenotype. This shift in macrophage polarization results in a significant prolongation of the pro-resolving effect (53). Notably, even after discontinuation of IL-4, a pro-relieving effect persists, indicating a critical role of macrophages in pain regulation (59). Multiple preclinical studies have demonstrated the pain-relieving effects of SPMs on various types of pathological pain. Macrophages express GPR37, a receptor for NPD1 (a type of SPM), and the absence of this receptor impairs the polarization of M2 macrophages, ultimately resulting in the failure of pain relief and the persistence of pain (54). Conversely, activation of macrophage GPR37 exerts a palliative effect on pain-like behavior (60). In summary, M2 macrophage polarization is a common mechanism by which multiple pro-pain relief mediators act. Therefore, the modulation of M2 macrophage polarization could be a promising strategy to alleviate pain.

## 5 Mechanisms of M2 macrophages in pain relief

### 5.1 IL-10

A study found that mice deficient in IL-10 or intrathecal administration of anti-IL-10 antibody have difficulty recovering from CIPN (61). Both T cells and macrophages can secrete IL-10. However, CD8^+^ T cells from IL-10^−/−^ mice retain their capability to promote the resolution of pain induced by paclitaxel, indicating that CD8^+^ T cells are not the source of IL-10 during pain relief, at least in this model (62). Meanwhile, CD8^+^ T cells can interact with macrophages *via* IL-13 to shift macrophages toward M2 (CD206^+^CD11c^–^) macrophages during the resolution of CIPN. This shift increases IL-10 production by macrophages (52).These studies suggest that M2 macrophages are the main source of IL-10 during the resolution of CIPN. Whether this conclusion remains valid in other models requires further research. CD163 on M2 macrophages mediates the production and secretion of IL-10 (50, 63). On the one hand, IL-10 can induce the macrophage itself to promote efferocytosis and inhibit neuroinflammation (64), which will be described in detail later. On the other hand, IL-10 directly interacts with IL-10R on sensory neurons to down-regulate the voltage-gated sodium channels in DRG, regulating spontaneous activity and depolarizing spontaneous fluctuations (65).

### 5.2 Opioid

Opioid peptides are endogenous neurotransmitters that exert analgesic effects by binding to opioid peptide receptors (66). They reduce the excitability of neurons and inhibit the transmission of pain signals, thereby relieving pain. Various types of leukocytes, including macrophages and T cells, are capable of producing opioid peptides (67–71). Among macrophages, M2 macrophages release higher levels of opioid peptides, such as Met-enkephalin, dynorphin, and β-endorphin, both *in vivo* and *in vitro*, compared to M1 and M0 macrophages (72). When these macrophages are transferred to injured sites, they alleviate pain hypersensitivity. However, this effect can be reversed by opioid receptor antagonists (72). Considerable research has shown that IL4, as an anti-inflammatory cytokine, has neuroprotective effects on the injured nervous system and can alleviate pain. The application of IL4 at the site of nerve injury can alleviate pain by polarizing macrophages to become M2 macrophages that release opioid peptides. Similarly, this effect can be reversed by opioid receptor antagonists (53).

### 5.3 Specialized pro-resolving mediators

M2 macrophages have been found to possess a higher proportion of pro-resolving mediators than pro-inflammatory mediators (73). In a study investigating the lipid mediator profiles of different subtypes of human macrophages, it was observed that M2 macrophages synthesize significantly higher levels of SPMs compared to M1 macrophages. These SPMs include D/E-series resolvins, protectins, maresins, and lipoxins (74). Notably, SPMs have demonstrated efficacy in alleviating various types of pain, including neuropathic, inflammatory, postoperative, and cancer pain (75, 76). SPMs exert their antinociceptive effects through the activation of G protein-coupled receptors (GPCRs) (77). These SPM receptors are widely expressed on sensory neurons and immune cells including macrophages, glial cells and neutrophils (76). SPMs play vital roles in promoting macrophage phagocytosis, suppressing microglia activation, preventing neutrophil recruitment, and inhibiting the release of inflammatory factors to alleviate pain (78–80). Meanwhile, SPMs potently inhibit TRPV1 and TRPA1 on nociceptive sensory neurons, thereby affecting excitatory synaptic transmission and pain signal transduction (80, 81).

### 5.4 Extracellular vesicles

Macrophages can produce a large number of extracellular vesicles (EVs) containing miRNAs, proteins, lipids, and many other biologically active substances, which contribute to several signaling events and physiological and pathological processes. Among them, miRNAs, short pieces of single-stranded RNAs (21-24 nucleotides), play important roles in the regulation of pain. Extensive studies have shown significant changes in miRNA expressions in the DRG and spinal cord following inflammatory pain and peripheral nerve injuries, including CFA, SNI, nerve crush, CCI, nerve transection, and spinal nerve ligation (82, 83). The altered miRNAs can be reversed by intrathecal injection of EVs derived from M2 macrophages (M2φ-Evs) that deliver miRNAs, leading to the recovery of pain threshold (83, 84). These functional miRNAs in EVs can be taken up by primary cortical neurons, microglia, and astrocytes, where the expression of pro-inflammatory miRNA target genes is downregulated (85).

### 5.5 Mitochondrial transfer

Due to their special structure, neurons have a greater energetic demand compared to other cell types (86). Approximately 66.7% of patients with mitochondrial disease are accompanied by chronic pain, often with a neuropathic nature (87). Disturbances in mitochondrial OXPHOS, oxidative stress and Ca2^+^ buffering are closely associated with both inflammatory and neuropathic pain, which might facilitate the development and maintenance of pain and drive the transition from acute pain to chronic pain (88–90). Several preclinical studies have been devoted to relieving pain by scavenging reactive oxygen species (91), inhibiting apoptotic pathways (92), altering mitochondrial membrane potential (91), or altering mitochondrial dynamics (93). However, these studies only aimed at improving specific aspects of mitochondrial dysfunction and its clinical translation has not been achieved so far. In contrast to M1 macrophages, the mitochondria of M2 macrophages are not dominated by glycolysis but by OXPHOS (47). During the physiological process of pain relief, M2 macrophages surrounding the sensory neurons in the DRG transfer mitochondria to sensory neurons through an interaction between the CD200R on M2 macrophages and the non-canonical CD200R-ligand iSec1 on sensory neurons. This transfer might be helpful for the recovery of mitochondria dysfunction containing OXPHOS and Ca2^+^ buffering in sensory neurons (52). Once the process is disrupted, pain resolution becomes challenging.

### 5.6 Efferocytosis

Efferocytosis is the process of phagocytosis of cellular debris by macrophages, especially M2 macrophages, after cell death or apoptosis, followed by cytokine release from macrophages (94). This process is crucial in inflammatory remission. During neuropathic pain, the expression of efferocytosis-related molecules, such as MerTK, on M2 macrophages is significantly downregulated at the site of injury compared to M2 macrophages at normal sites. Consequently, efferocytosis is significantly deficient, and dead or dying cells cannot be completely cleared (95). It was also found that the efferocytosis of synovial macrophages in OA patients was markedly reduced compared to that in healthy individuals (96). However, 3,3’-diindolylmethane (DIM) enhances macrophage efferocytosis, leading to the subsequent relief of visceral pain. And inhibition of macrophage efferocytosis reversed the pain-relieving effect of 3,3’-diindolylmethane (97). This implies that inadequate efferocytosis of M2-like macrophages plays a crucial role in the development of chronic inflammation in damaged nerves and reflects the critical role of normal efferocytosis of M2-like macrophages in the pain relief process.

### 5.7 Increase in barrier stability

The blood-spinal cord barrier (BSCB) is formed by tightly connected capillary endothelial cells, basal laminae, pericytes and astrocyte peduncles around neurons. It serves as a bridge mediating the interaction between the immune and nervous systems (98). The tight junctions between the endothelial cells of the BSCB create a physical barrier that separates the blood from the spinal cord (99), where claudin-1, claudin-5, occluding and ZO-1 play important roles in maintaining the integrity of the BSCB (100). It prevents the influx of blood cells and neurotoxic substances into the spinal cord, thereby playing a crucial role in maintaining the stability of the perineuronal environment and ensuring normal neuronal function (99). Pain induced by peripheral neuropathy is often accompanied by the disruption of BSCB integrity (100–102). Inflammatory factors and immune cells, including T cells, invade around spinal cord neurons, thereby inducing nociceptive hyperalgesia. Recent research has shown that M2 macrophages can help restore BSCB integrity by secreting large amounts of TGF-β, which binds to receptors on endothelial and pericytes, leading to the upregulation of ZO-1, occludin, and N-cadherin (103). On the other hand, endothelial cells can also promote M2 macrophage polarization by releasing lactic acid (104). Besides the BSCB, the blood-nerve barrier and blood-DRG barrier also play an important role in pain maintenance (105). In the diabetic neuropathic pain model, the content of vascular-associated macrophages was significantly down-regulated (105). To some extent, this also reflects the role of macrophages in maintaining barrier integrity during pain relief. However, further research in this area is needed.

## 6 Discussion and conclusion

**Figure 1:**
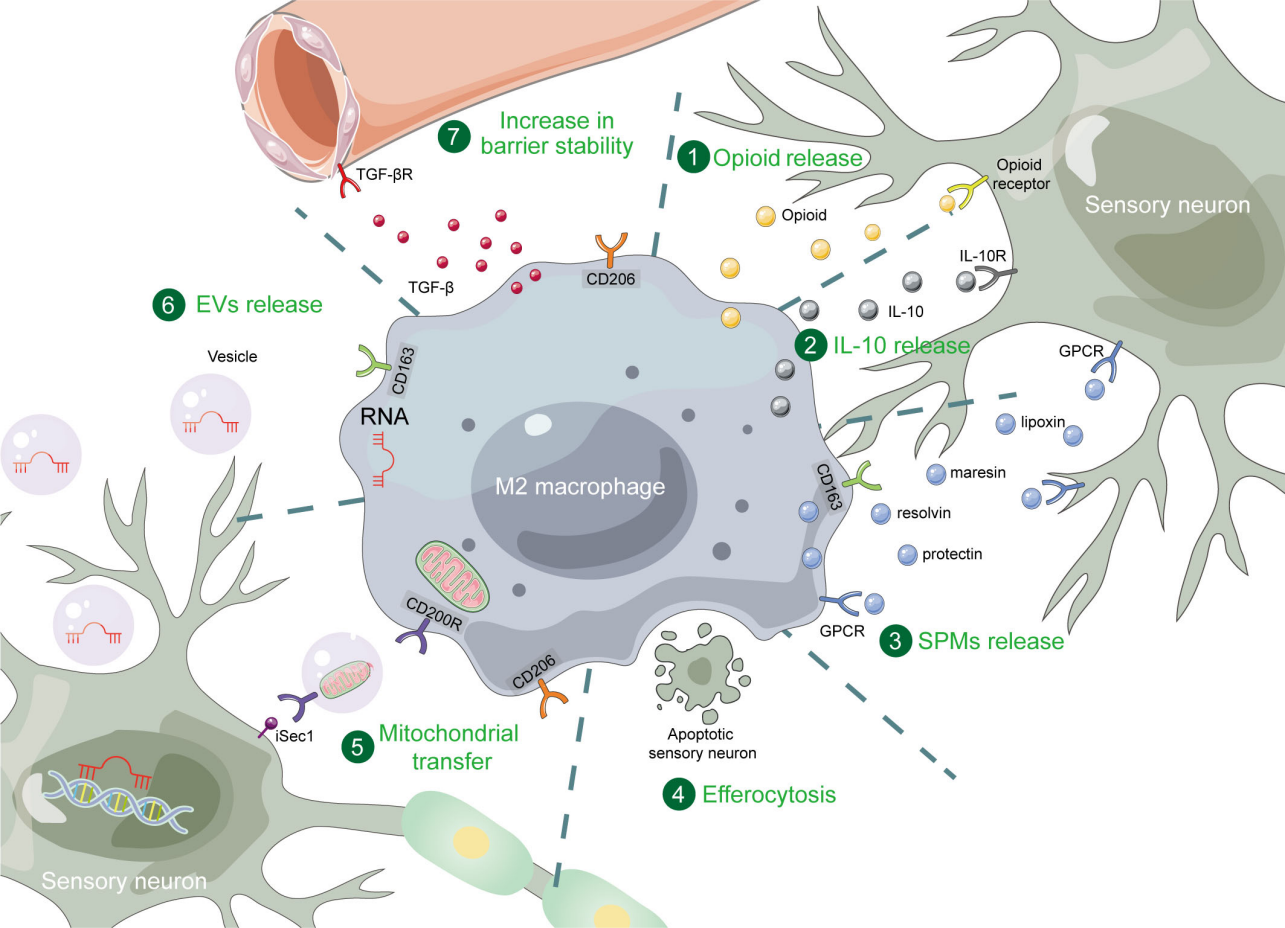
The mechanisms of M2 macrophages in pain relief. M2 macrophages exert pain relief through opioid, IL-10, SPMs, efferocytosis, mitochondrial transfer, EVs, and increase in barrier stability. SPM, specialized pro-resolving mediator; GPCR, G protein-coupled receptor; EV, extracellular vesicle.

As crucial components of the immune system, macrophages play significant roles in the process of pain modulation. In recent years, more and more studies have focused on the role of M2 macrophages in pain relief. In this review, we have summarized the regulation of M2 macrophages and their important role in pain relief. M2 macrophage polarization is primarily associated with JAK-STATs, TGF-β and PPARγ signaling pathways, and multiple metabolic pathways. Targeting these pathways can modulate M2 macrophage polarization. In the process of pain relief, the number of M2 macrophages increased significantly. Manipulating M2 macrophages can also relieve pain. These polarized M2 macrophages exert their effects through various direct or indirect mechanisms. Firstly, M2 macrophages release substances such as IL-10, opioids, and SPMs, which can act on the relevant receptors on the nociceptive sensory neurons. Additionally, M2 macrophages transfer their miRNAs and mitochondria to nociceptive sensory neurons *via* EVs. Moreover, they can phagocytose apoptotic sensory neurons through efferocytosis and enhance vascular stability, thus providing a favorable environment for neurons and contributing to pain relief (see **Figure 1** for summary).

However, several unresolved issues remain. M1 macrophages play indispensable roles in the development of pain following injury, and the failure of macrophages to transition from the M1 to M2 phenotype may contribute to the persistence of acute pain and its progression into chronic pain. Of course, it would be imprudent to block the bactericidal and phagocytic effects of M1 macrophages as they are required in the early stages of injury (106). Therefore, further exploration is needed to determine the optimal timing for M2 macrophage polarization. Besides, the process of M2 macrophages polarization is dynamic, which is tissue, injury and time-dependent. It is not rigorous to infer the mechanism of M2 macrophages polarization in one injury or tissue type from that in another injury or tissue type. Therefore, it is necessary to study macrophages in a spatiotemporal-specific manner. Last, several studies have confirmed that efferocytosis is one of the most vital mechanisms in M2-mediated pain relief. But the role played by the molecules involved in efferocytosis or other scavenger receptors has hardly been studied. Gas6, one of the molecules involved in efferocytosis, has been reported to regulate efferocytosis during obesity-related OA development (107). OA is closely related to pain (108). We speculate that Gas6 is involved in M2-mediated pain relief through regulating efferocytosis. Therefore, the molecules involved in efferocytosis or other scavenger receptors may also play important roles in M2-mediated pain relief, which is also the direction for the further research.

Pain often coexists as a symptom of many diseases (109–111), and numerous studies have demonstrated that M2 macrophages play a positive role in alleviating symptoms of various diseases, such as rheumatoid arthritis and OA (112–114). By deepening our understanding of the mechanisms through which M2 macrophages promote pain relief, we can develop novel strategies for pain management that not only provide symptomatic relief but also actively address the underlying causes of pain, achieving a dual effect of treating both symptoms and root causes.

In conclusion, M2 macrophages have a positive impact on pain relief, and targeting the regulation of M2 macrophage polarization holds promise as an effective approach for pain relief.

## Author contributions

WZ and LM wrote the manuscript; DD, TZ, LH, FX, SH and YD contributed to literature collection and discussion; XC edited the manuscript. All of the authors have approved the final manuscript.
